# The Risk Factors of Child Lead Poisoning in China: A Meta-Analysis

**DOI:** 10.3390/ijerph13030296

**Published:** 2016-03-08

**Authors:** You Li, Jian Qin, Xiao Wei, Chunhong Li, Jian Wang, Meiyu Jiang, Xue Liang, Tianlong Xia, Zhiyong Zhang

**Affiliations:** Department of Occupational and Environmental Health, School of Public Health, Guangxi Medical University, Shuangyong Road No.22, Nanning 530021, China; liyou121300@163.com (Y.L.); gxmucommoner@163.com (J.Q.); gxmu_xwei@126.com (X.W.); lichunhong@gxmu.edu.cn (C.L.); wangjian2007@hotmail.com (J.W.); 13481022184@163.com (M.J.); 490275305@139.com (X.L.) xiatianlong@126.com (T.X.)

**Keywords:** children, lead poisoning, risk factors, meta-analysis, China

## Abstract

*Background:* To investigate the risk factors of child lead poisoning in China. *Methods:* A document retrieval was performed using MeSH (Medical subject heading terms) and key words. The Newcastle-Ottawa Scale (NOS) was used to assess the quality of the studies, and the pooled odd ratios with a 95% confidence interval were used to identify the risk factors. We employed Review Manager 5.2 and Stata 10.0 to analyze the data. Heterogeneity was assessed by both the Chi-square and *I^2^* tests, and publication bias was evaluated using a funnel plot and Egger’s test. *Results:* Thirty-four articles reporting 13,587 lead-poisoned children met the inclusion criteria. Unhealthy lifestyle and behaviors, environmental pollution around the home and potential for parents’ occupational exposure to lead were risk factors of child lead poisoning in the pooled analyses. Our assessments yielded no severe publication biases. *Conclusions:* Seventeen risk factors are associated with child lead poisoning, which can be used to identify high-risk children. Health education and promotion campaigns should be designed in order to minimize or prevent child lead poisoning in China.

## 1. Introduction

Lead has been impacting human health since the Romans began mining it over 2500 years ago [1]. Lead widely exists in the environment and can pollute the food chain, soil, water and air, leading to human diseases [2]. Lead exposure likely impairs motor function [3], and negatively impacts intellectual development, hemoglobin formation and childhood growth [4]. Many human activities such as home painting; smoking; using leaded petrol; eating foods contaminated with lead like popcorn, canned food and preserved eggs; drinking from leaded water pipes; smelting; and especially industry manufacturing processes are associated with lead exposure [5]. Children, who are still growing, are more likely to be sensitive to the harmful effects of lead [6]. The diagnostic criteria for child lead poisoning have been revised in some developed countries [7,8]. Currently, the Centers for Disease Control and Prevention in the U.S. recommend a reference level of five micrograms per deciliter to identify children with blood lead levels. However, we used the recommendation (10 μg/dL) in the light of included studies of our meta-analysis.

Though the number of child lead poisoning cases has decreased during recent decades in some countries [9,10], lead poisoning still remains an important public health issue for tens of millions of children in the world, especially in developing countries [11,12,13], including China.

Although numerous studies have identified the risk factors of child lead poisoning, inconsistency has been presented for different risk factors among various study populations. On account of the increasing importance of the identification of high-risk children, this meta-analysis was conducted with the objective of investigating the overall risk factors of child lead poisoning in China.

## 2. Methods

### 2.1. Literature Selection

Two reviewers independently identified relevant studies published in English and Chinese from January 1980 to October 2015 by searching the following electronic databases: PubMed, EMBASE, the Cochrane Library, Science Citation Index, Chinese biomedical literature database (CBM), Wangfang database, VIP database and China National Knowledge Infrastructure (CNKI). The following keywords were used: ((lead OR Pb or blood lead OR lead poisoning) AND (children) AND (risk factors OR influence factors) AND (China)). These studies were included in this meta-analysis if: (1) blood lead level was up to 100 μg/L (or more than 10 μg/dL or 4.83 μmol/L) for lead poisoning; (2) there were sufficient data to calculate the odds ratio (OR) and 95% confidence interval (CI); (3) the study followed a case-control or cohort design; (4) at least one risk factor was identified as being associated with lead poisoning; and (5) the full text was available. Studies were excluded if: (1) they did not comply with the requirements listed above; (2) did not study the relevant research population group or its studied populations had overlaps; (3) had incomplete information or abnormal data; or (4) were themselves reviews. The discrepancies were resolved by discussion.

### 2.2. Data Extraction

Data were extracted from each included study by two reviewers, separately. The following data were recorded: (1) name of the first author; (2) year of publication; (3) numbers in case and control groups; (4) name of journal; and (5) the study site. Additional information was extracted when required.

### 2.3. Quality Assessment

The Newcastle-Ottawa Scale (NOS) for case-control study was used to evaluate the quality of our studies [14]. The criteria included three categories: (1) selection (4 items); (2) comparability (1 item); and (3) exposure for case-control study (2 items). A study was awarded a maximum of one star for each item, with the exception of comparability for which two stars were given. Articles with more than seven stars were considered to be of a high quality; those with four to six stars were considered as moderate quality studies; and of a poor quality if they had fewer than four stars.

### 2.4. Statistical Analysis

The statistical analysis was conducted using Review Manager 5.2 (Cochrane Collaboration, Oxford, UK) and Stata version 10.0 (Stata Corporation, College Station, TX, USA). The results were reported as pooled odds ratios (ORs) and corresponding 95% confidence intervals (CI). A two-sided *p* < 0.05 was considered statistically significant. The heterogeneity of the included studies was evaluated using the Cochran’s *Q* test and the *I*^2^ test [15]. *I*^2^ is the proportion of total variation attributable to between-study heterogeneity as opposed to random error or chance, and *I*^2^ values of 25%, 50% and 75% are considered to indicate low, moderate, and high heterogeneity, respectively. Commonly, we selected a random-effects model to calculate corresponding parameters when the *I*^2^ value was more than 50%. Otherwise, a fixed-effects model was used. A funnel plot (Review Manager 5.2) and Egger’s test [16] (Stata 10.0) were used to assess publication bias—the statistical publication bias was set at *p* < 0.10. The funnel plot and Egger’s test were not used to analyze publication bias when less than 5 studies were available [17]. For these data, we checked the outlier studies and analyzed the reasons for the abnormal values.

## 3. Results

### 3.1. Identification of Selected Studies

**Figure 1:**
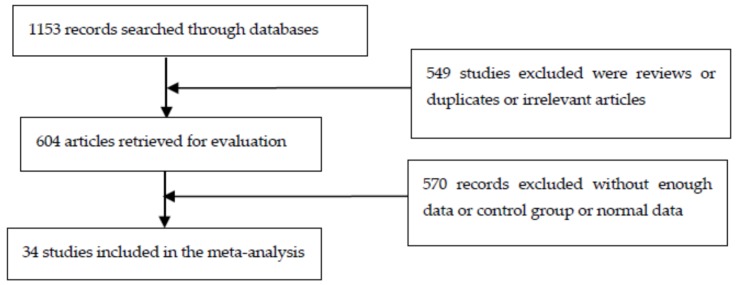
Flow chart of study selection.

A total of 1153 articles were obtained from the online databases. Five hundred and forty-nine studies were excluded because they were reviews, duplicates or irrelevant studies, and 604 studies were retained. However, 570 of them lacked the necessary data, a control group or normal data, so they were excluded. Eventually, 34 papers [18,19,20,21,22,23,24,25,26,27,28,29,30,31,32,33,34,35,36,37,38,39,40,41,42,43,44,45,46,47,48,49,50,51] were entered into the final meta-analysis, as shown in Figure 1. Of these, 12 articles earned eight stars, 15 others obtained seven stars, and the remaining seven papers were awarded six stars. Hence, the included studies were of a relatively high quality.

### 3.2. Study Characteristics

Thirty-four studies from January 1980 to October 2015 with 13,587 lead-poisoning cases were enrolled. The basic characteristics of these studies are summarized in Table 1.

### 3.3. Assessment of Heterogeneity

Heterogeneities were discovered in the distribution for the between-study variance of these factors: home painting; living near main roads; passive smoking; often eating foods containing lead; frequent consumption of dairy products; potential for father’s occupational exposure to lead; potential for mother’s occupational exposure to lead; sex; industry around the home; hand-to-mouth activity; living on the ground floor of a building; coal burning; daily intake of calcium, iron, and/or zinc supplements; mother’s educational level; father’s educational level; often not washing hands at key times; picky eating; and peeling walls in the living quarters. Therefore, we calculated the pooled OR values, using a random-effects model, and the results are listed in Table 2.

### 3.4. Risk Factors

The review identified 30 risk factors associated with child lead poisoning from the 34 studies. The ORs, along with their 95%CIs, of the risk factors are presented in Table 3. Of these, 12 factors were examined in only one or two studies, and the remaining 18 factors were included in three or more studies. We were then able to estimate the pooled ORs for these 18 factors. The results of the pooled analysis are displayed in Table 3. The forest plots for the risk factors are shown in Figure S1.

### 3.5. Publication Bias

We conducted the Egger’s test and funnel plots to evaluate potential publication bias. The results of the Egger’s test (shown in Table 4) suggested that the publication biases among all of the risk factors were not statistically significant (*p* > 0.10).The visual inspection of the funnel plots (Figure S2)—narrow tops and wide bottoms—indicated no significant asymmetry. Hence, our investigation indicated no severe publication biases.

## 4. Discussion

This research makes an attempt to sum up the findings concerning the risk factors for child lead poisoning in China during the last three decades. Thirty-four studies eventually met the study criteria and were included into our meta-analysis. Some of these risk factors were among different studies. The pooled analysis from our findings confirmed that children faced a lower likelihood of experiencing lead poisoning if they live home painting, take daily supplements of calcium, iron and zinc, have a father or mother of a higher educational level. Factors such as living near main roads, passive smoking, often eating foods containing lead, potential for parents’ occupational exposure to lead, sex, industry around the home, hand-to-mouth activity, often not washing hands at key times, picky eating, living on the ground floor of a building, coal burning and peeling walls in the living quarters were associated with an increased likelihood of being lead poisoned. Our findings are in line with those of a previous study in China [52]. Lead poisoning not only has a negative effect on the development of children’s intelligence and behavior, but it also leads to anemia and can even cause death [53]. The risks of lead poisoning are multifactorial; therefore, future study is needed in order to explore on how the risk factors interact with each other.

### 4.1. Home Painting and Peeling Walls

Lead-based paint is a well-established cause of lead poisoning among children. The most common lead materials are paint and coating used in home decorations. In our study the OR of home painting was less than 1, which meant that home painting showed a beneficial effect on children’s health. This may be due to the fact that Xiulan Ma [43], Huiyan Liu [35] and MeilinPeng’s [40] articles had a higher percentage of home painting in the control group than the lead-poisoning group. Whether leaded paint was used or not was not mentioned in those articles, so we could not analyze the data for home painting separately. Consequently, the meta-analysis requires more studies to verify home painting as a risk factor. Meanwhile, peeling walls contain lead, e.g., in the paint and/or pigment; thus, children are more likely to contact lead under such circumstances. Consequently, peeling walls is one of the risk factors for child lead poisoning.

### 4.2. Living Near Main Roads

Previous studies have consistently documented that the residential location near main traffic roads is a vital variable in the effect of traffic-related air pollution on health [54,55]. Although leaded gasoline has been banned for use in China since 1 July 2000, a small amount of lead can still be found in crude leaded oil and so it is also in gasoline. Due to the widespread use of leaded gasoline before, the lead from automobile exhaust was deposited in water, soil and in different species [56], and finally absorbed by children. Therefore, automobile exhaust is an important lead pollution source, especially in heavy-traffic populated areas [57]. Our findings revealed that living near main roads is a risk factor for child lead poisoning. Due to living near main roads children have more opportunities to contact lead, in the air they breathe and at the same time in the polluted foods by automobile exhaust they eat. Additionally, living near main roads is not only related to the occurrence of lead poisoning but also to allergic diseases [58].

### 4.3. Passive Smoking

China is the largest producer and consumer of tobacco globally. According to the data, over 350 million smokers [59], 740 million passive smokers and 180 million children under the age of 15 live in China [60,61]. While cigarettes contain heavy metals such as cadmium and lead, second-hand smoking exposure can increase the level of lead in child blood. A recent study found that parental smoking at home is associated with an elevated level of lead in the blood [62]. Moreover, second-hand smoke can cause the respiratory diseases like chronic cough or asthma in children [63,64]. The present study revealed that the children with lead poisoning were exposed to more passive smoking compared with the control children.

### 4.4. Often Eating Foods Containing Lead

Our meta-analysis showed that eating foods containing lead often is a risk factor for child lead poisoning (OR=2.99, 95%CI: 1.95, 4.59). Among the foods containing lead were popcorn, preserved eggs, fried potato chips, spoiled traditional Chinese moon cakes, canned food and seafood. As traditional popcorn machines in China are made from lead alloy and high temperature can release lead vapors, the lead levels in Chinese traditionally-made popcorn can be elevated.

### 4.5. Potential for Parents’ Occupational Exposure to Lead

Childhood family members of workers engaged in a lead-related occupation are considered to be at additional risk of child lead poisoning. Such parents tend to pay little or no attention to carrying lead dust from the workplace to their household.

Our investigation suggested that the parents’ occupational exposure to lead is a risk factor for lead poisoning among children.

### 4.6. Mother’s or Father’s Educational Levels

Parental educational levels have a great effect on the healthy growth of children [65]. Maternal or paternal educational level, for example, can indirectly affect the children’s blood lead concentration. We found that a mother (OR = 0.66, 95%CI: 0.63, 0.70) or father (OR = 0.54, 95%CI: 0.46, 0.63) with an educational attainment level above a high school degree was a protective factor against child lead poisoning. The higher the mother’s and/or father’s educational level, the more attention they tend to the healthy growth of children; hence, the chance of the children of such parents to contact lead is relatively smaller, and the incidence them suffering lead poisoning is lower than that of their counterparts.

### 4.7. Sex

The pooled analysis of 21 studies indicated that boys were more likely to develop lead poisoning than girls, which was in agreement with the findings of the majority of the included papers. Nevertheless, we did not analyze various ages as a risk factor because there were differences in the age segmentations between the papers.

### 4.8. Industry Around the Home

The pollution of lead mainly comes from the environment and often involves industrial smelting, manufacturing and other related industrial and mining enterprises.

The lead dust sediments on the ground in the form of granules or suspends in the air with gel. A number of industries, including those dealing with battery manufacturing, metal smelting, printing, mechanical manufacturing and shipbuilding are found in China. Thus, it is possible for the lead dust to contaminate local residents. The data show that industry around the home is a risk factor that can give rise to child lead poisoning.

### 4.9. Hand-to-Mouth Activity, Often Not Washing Hands at Key Times

Our findings indicated that hand-to-mouth activity and often not washing hands at key times are risk factors that can lead to child lead poisoning among children. These behaviors not only influence one’s health but also increase the burdens of both the family and the nation [66]. For example, not handwashing with soap at key times not only increase diarrheal disease and acute respiratory infection [67,68,69], but it may also lead to chronic lead poisoning. What is more, the behaviors developed during childhood can affect one’s health during both youth and adulthood. Thus, it is important to help children establish healthy lifestyles and behaviors before the unhealthy ones are firmly developed.

### 4.10. Picky Eating, Daily Intake of Calcium Iron Zinc Supplements

Picky eating among children is a public health issue in China. During childhood, children are rapidly growing and need more nutrients. Therefore, their dietary habits are critical to their physical development. Picky eaters often consume a small amount of food and may be prone to deficiencies in trace elements more easily, potentially impacting their childhood growth [70,71,72]. Furthermore, deficiencies in trace elements like zinc, iron, calcium, copper and so on in the diet could increase the absorption of lead [73,74].Evidence from our study showed that picky eating is a risk factor, and that the daily intake of calcium, iron, and zinc supplements is a protective factor against child lead poisoning. Therefore, it is important for parents to instruct their children to have a healthy diet.

### 4.11. Living on the Ground Floor of a Building

Living on the ground floor may facilitate the contact with lead dust and smoke. Lead floating in the air and in the soil can enter the living quarters of lower floors with more ease than those living on higher floors, which leads to the children of such families having a higher rate of lead poisoning occurrence. Our meta-analysis showed that living on the ground floor is a risk factor for child lead poisoning.

### 4.12. Coal Burning

The use of solid fuels is a significant public health concern. With the rapid economic expansion and industrial growth, demand for energy is increasing in China. It relies on coal for about 70%–75% of its energy needs [75]. The smoke of burning coal contains polycyclic aromatic hydrocarbons, fine particles, sulfur dioxide, carbon monoxide, lead, and other harmful matter [76]. Coal burning can release lead and mercury and other heavy metals into the atmosphere [77,78,79]. Thus, in China, it has resulted in massive amounts of environmental pollution. Our results indicated that burning coal at home is a risk factor for child lead poisoning, so we should decrease the burning of coal at home in order to protect children’s health.

## 5. Conclusions

The evidence from this review indicates that 17 risk factors are associated with child lead poisoning in China, which can be used to identify high-risk children. Health education and promotion campaigns that aim to tackle challenges like correcting bad habits in children, paying attention to children’s personal hygiene and strengthening nutrition guidance should be designed and implemented in order to minimize or prevent the occurrence of lead poisoning among children in China.

## Figures and Tables

**Table 1:** Basic characteristics of the studies in this meta-analysis.

No.	Year of Publication	First Author	*Journal*	Total Lead-Poisoning Cases (*n*)	Total Controls (*n*)	Research Site
1	2001	Qi Ye	*Journal of Huaihai Medicine*	9	91	Lianyungang
2	2003	Xizheng Ouyang	*Practical Preventive Medicine*	301	823	Shaoyang
3	2003	Xinxin Chen	*Zhonghua Liu Xing Bing Xue Za Zhi*	807	1455	Beijing
4	2004	Qingrong Zhang	*Maternal and Child Health Care of China*	600	412	Guangzhou
5	2004	Sun Li	*Int. J. Hyg. Environ. Health*	63	154	Zhejiang
6	2004	Hongzhong Zhang	*Huazhong University of Science and Technology*	418	120	Zhuhai
7	2005	Xiping Ma	*Studiesof Trace Elements and Health*	316	702	Pingdingshan
8	2005	Yan Yi	*Maternal and Child Health Care of China*	69	61	Shiyan
9	2006	Xiaozhen Xiao	*Guangdong Trace Elements Science*	41	161	Huizhou
10	2006	Hong Tian	*J. Appl. Clin. Pediatr.*	459	596	Beijing
11	2006	Aifang Huang	*Zhejiang Prev. Med.*	320	529	Yuhuan
12	2006	Bin He	*Practical Preventive Medicine*	87	1085	Changsha
13	2006	Shuang Yang	*Dalian Medical University*	30	143	Dalian
14	2006	Sulin Fu	*Anhui J. Prev. Med.*	92	919	Hefei
15	2006	Xianxiang Feng	*Journal of Youjiang Medical College For Nationalities*	764	1187	Liuzhou
16	2007	Jiazheng Xu	*China Tropical Medicine*	274	1997	Haikou
17	2007	Shuwei Zhang	*China Tropical Medicine*	523	1193	Jining
18	2007	Huiyan Liu	*Huazhong University of Science and Technology*	83	1518	Wuhan
19	2008	Xiaohua Liu	*Journal of Zhengzhou University(Medical Sciences)*	120	27	Xinxiang
20	2010	Shiqiong Wang	*Journal of Jianghan University (Natural Sciences)*	96	197	Wuhan
21	2010	Ailan Gou	*Maternal and Child Health Care of China*	158	158	Nanping
22	2011	Guiping Chang	*Journal of Yangtze University (Natural Science Edition)*	42	415	Jingzhou
23	2011	Jie Shan	*Prev. Med. Trib.*	233	3990	Weifang
24	2011	Meilin Peng	*Chinese Journal of Child Health Care*	51	3343	Hefei
25	2011	Zangwen Tan	*Chin. J. Pediatr.*	5295	64,673	China
26	2012	Xiulan Ma	*Chinese Journal of Healthy Birth & Child Care*	153	1357	Lanzhou
27	2012	Jianghong Liu	*Paediatr Perinat Epidemiol*	105	1239	Jintan
28	2013	Yinyun Long	*Journal of Chinese Physician*	239	5612	Changde
29	2013	Xiaofeng Gao	*Maternal and Child Health Care of China*	38	263	Tangshan
30	2014	Shengliang Sun	*Chin. Med. J. Metall. Indus.*	101	101	Dalian
31	2014	Pi Guo	*PloS ONE*	165	658	Shantou
32	2014	Zhenyan Gao	*Suzhou University*	179	1849	Taizhou
33	2015	Zhong Chen	*Chinese Journal of Child Health Care*	592	7590	Wuhan
34	2015	Ning Jin	*Chinese Journal of Modern Drug Application*	764	236	Shanxi

**Table 2:** Results of heterogeneity test using random-effects and fixed-effects models.

Risk Factors	Paper Number	χ^2^	*p*	*I^2^* (%)	Meta Analytical Model
Home painting	13	100.75	<0.00001	88	random
Living near main roads	12	77.72	<0.00001	86	random
Passive smoking	12	103.17	<0.00001	89	random
Often eating foods containing lead	22	581.00	<0.00001	96	random
Frequent consumption of dairy products	6	14.67	0.01	66	random
Daily intake of calcium, iron, and/or zinc supplements	4	5.23	0.16	43	fixed
Potential for father’s occupational exposure to lead	16	97.60	<0.0001	85	random
Potential for mother’s occupational exposure to lead	8	34.52	<0.0001	80	random
Mother’s educational level	11	12.95	0.23	23	fixed
Father’s educational level	7	10.21	0.12	41	fixed
Sex	21	42.70	0.002	53	random
Industry around the home	7	32.10	<0.0001	81	random
Hand-to-mouth activity	14	83.04	<0.00001	84	random
Often not washing hands at key times	10	15.91	0.07	43	fixed
Picky eating	7	7.52	0.28	20	fixed
Living on the ground floor	9	114.04	<0.00001	93	random
Coal burning	6	15.68	0.008	68	random
Peeling walls	3	3.79	0.15	47	fixed

**Table 3:** Meta-analysis of the risk factors for child lead poisoning in China.

Risk Factors	Number of Papers	Total Lead-Poisoning Cases (*n*)	Total Control Cases (*n*)	OR (95% CI )	*Z*	*p*
Home painting	13	2337	9963	0.12 (0.05, 0.19)	3.24	0.001
Living near main roads	12	2449	13,358	2.22 (1.53, 3.22)	4.22	<0.0001
Passive smoking	12	2209	7476	2.10 (1.38, 3.20)	3.46	0.0005
Often eating foods containing lead	22	10,320	90,875	2.99 (1.95, 4.59)	5.03	<0.00001
Frequent consumption of dairy products	6	6512	72,047	0.84 (0.69, 1.01)	1.88	0.06
Daily intake of calcium, iron, and/or zinc supplements	4	6083	74,212	0.80 (0.74, 0.86)	6.07	<0.00001
Potential for father’s occupational exposure to lead	16	2549	15,401	2.26 (1.62, 3.15)	4.81	<0.00001
Potential for mother’s occupational exposure to lead	8	1359	13,566	1.53 (1.04, 2.26)	2.15	0.03
Mother’s educational level	11	7273	76,429	0.66 (0.63, 0.70)	15.38	<0.00001
Father’s educational level	7	1538	9640	0.54 (0.46, 0.63)	7.84	<0.00001
Sex	21	5263	23,536	1.39 (1.24, 1.55)	5.66	<0.00001
Industry around the home	7	6203	70,253	1.67 (1.25, 2.22)	3.51	0.0005
Hand-to-mouth activity	14	7329	73,958	1.68 (1.29, 2.17)	3.91	<0.0001
Often not washing hands at key times	10	6480	74,330	1.22 (1.16, 1.29)	7.09	<0.00001
Picky eating	7	1601	4315	2.62 (2.15, 3.20)	9.49	<0.00001
Living on the ground floor	9	7658	82,256	1.58 (1.14, 2.18)	2.77	0.006
Coal burning	6	1705	10,597	1.45 (1.04, 2.02)	2.18	0.03
Peeling walls	3	5655	67,729	1.17 (1.09, 1.25)	4.55	<0.00001

**Table 4:** Publication bias of the risk factors for child lead poisoning in China.

Risk Factors	Paper Number	Egger’s Test (*p*-Value)
Home painting	13	0.199
Living near main roads	12	0.215
Passive smoking	12	0.109
Often eating foods containing lead	22	0.777
Frequent consumption of dairy products	6	0.755
Potential for father’s occupational exposure to lead	16	0.142
Potential for mother’s occupational exposure to lead	8	0.111
Mother’s educational level	11	0.135
Father’s educational level	7	0.335
Sex	21	0.834
Industry around the home	7	0.310
Hand-to-mouth activity	14	0.143
Often not washing hands at key times	10	0.152
Picky eating	7	0.229
Living on the ground floor	9	0.372
Coal burning	6	0.260

## Supplementary Materials

The following are available online at www.mdpi.com/www.mdpi.com/1660-4601/13/3/296/s1, Figure S1: Forest plots for risk factors of children’s lead poisoning.(**a**)home painting; (**b**) living near main roads; (**c**) passive smoking; (**d**) often eating foods containing lead; (**e**) frequent consumption of dairy products; (**f**) daily taking calcium, iron, zinc supplements; (**g**) potential for father's occupational exposure to lead; (**h**) potential for mother's occupational exposure to lead; (**i**) mother's educational level; (**j**) father's educational level; (**k**) sex; (**l**) industry around the home; (**m**) hand-to-mouth activity; (**n**) no often washing hands at key times; (**o**) picky eating; (**p**) living on the ground floor; (**q**) coal burning; (**r**) peeling walls. Figure S2. Funnel plots of risk factors for children’s lead poisoning. (**a**) home painting—living near main roads; (**b**) passive smoking—often eating foods containing lead; (**c**) frequent consumption of dairy products—potential for father's occupational exposure to lead; (**d**) potential for mother's occupational exposure to lead—father's educational level; (**e**) mother's educational level—sex; (**f**) industry around the home—hand-to-mouth activity; (**g**) no often washing hands at key times—picky eating; (**h**) living on the ground floor—coal burning.
