# Supplementary material for: The Risk Factors of Child Lead Poisoning in China: A Meta-Analysis
Source: Int J Environ Res Public Health. 2016 Mar 8;13(3):296. doi: 10.3390/ijerph13030296 (PMC4808959; doi:10.3390/ijerph13030296)
Supplement: Supplementary file 1 [file ijerph-13-00296-s001.pdf]

SupplementaryMaterials: The Risk Factors of Child Lead Poisoning in China: A Meta-Analysis

You Li, Jian Qin, Xiao Wei, Chunhong Li, Jian Wang, Meiyu Jiang, Xue Liang, Tianlong Xia and Zhiyong Zhang *

Study or Subgroup	Experimental Events	Experimental Total	Control Events	Control Total	Weight	Risk Difference M-H, Random, 95% CI
hong tian 2006	294	459	320	596	9.3%	0.10 [0.04, 0.16]
huiyan liu 2007	69	83	1320	1518	8.8%	-0.04 [-0.12, 0.04]
meilin peng 2011	39	51	2524	3341	7.8%	0.01 [-0.11, 0.13]
qi ye 2001	4	9	10	91	3.1%	0.33 [0.00, 0.67]
shengliang sun 2014	41	101	18	101	7.7%	0.23 [0.11, 0.35]
shiqiong wang 2010	62	96	56	197	7.9%	0.36 [0.25, 0.48]
sun li 2004	4	63	8	154	9.1%	0.01 [-0.06, 0.08]
xiaofeng gao 2013	19	38	21	263	6.5%	0.42 [0.26, 0.58]
xiaohua liu 2008	75	120	10	27	5.5%	0.25 [0.05, 0.46]
xiaozhen xiao 2006	23	41	51	161	6.4%	0.24 [0.08, 0.41]
xinxin chen 2003	613	807	1038	1455	9.7%	0.05 [0.01, 0.08]
xiping ma 2005	70	316	128	702	9.4%	0.04 [-0.01, 0.09]
xiulan ma 2012	65	153	783	1357	8.8%	-0.15 [-0.23, -0.07]
**Total (95% CI)**		**2337**		**9963**	**100.0%**	**0.12 [0.05, 0.19]**
Total events	1378		6287			

Heterogeneity: Tau² = 0.01; Chi² = 100.75, df = 12 (P < 0.00001); I² = 88%
Test for overall effect: Z = 3.24 (P = 0.001)

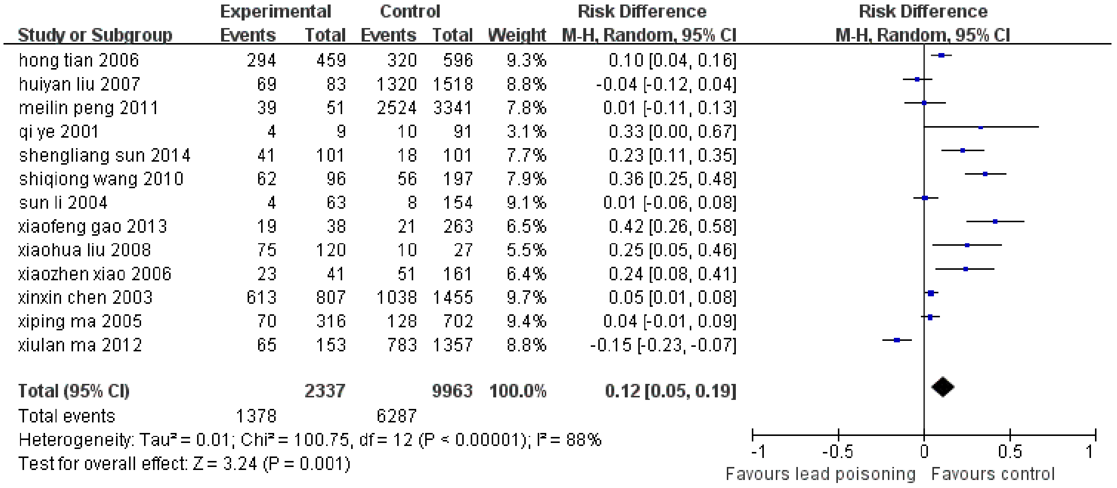

(**a**)

Study or Subgroup	Experimental Events	Experimental Total	Control Events	Control Total	Weight	Odds Ratio M-H, Random, 95% CI
aifang huang 2006	52	320	3	529	5.2%	34.02 [10.53, 109.94]
bin he 2006	14	87	149	1085	8.6%	1.20 [0.66, 2.19]
guiping chang 2011	18	42	72	425	8.2%	3.68 [1.90, 7.13]
huiyan liu 2007	20	77	358	1461	9.1%	1.08 [0.64, 1.82]
jie shan 2011	54	233	693	3990	10.2%	1.44 [1.05, 1.97]
meilin peng 2011	22	51	1492	3340	8.8%	0.94 [0.54, 1.64]
qinrong zhang 2004	322	600	138	412	10.5%	2.30 [1.77, 2.98]
shengliang sun 2014	41	101	18	101	8.3%	3.15 [1.65, 6.01]
shuang yang 2006	12	30	29	143	7.1%	2.62 [1.14, 6.05]
sun li 2004	57	63	140	154	6.1%	0.95 [0.35, 2.59]
xiaofeng gao 2013	17	38	18	263	7.3%	11.02 [4.96, 24.49]
xinxin chen 2003	194	807	279	1455	10.7%	1.33 [1.08, 1.64]
**Total (95% CI)**		**2449**		**13358**	**100.0%**	**2.22 [1.53, 3.22]**
Total events	823		3389			

Heterogeneity: Tau² = 0.32; Chi² = 77.72, df = 11 (P < 0.00001); I² = 86%
Test for overall effect: Z = 4.22 (P < 0.0001)

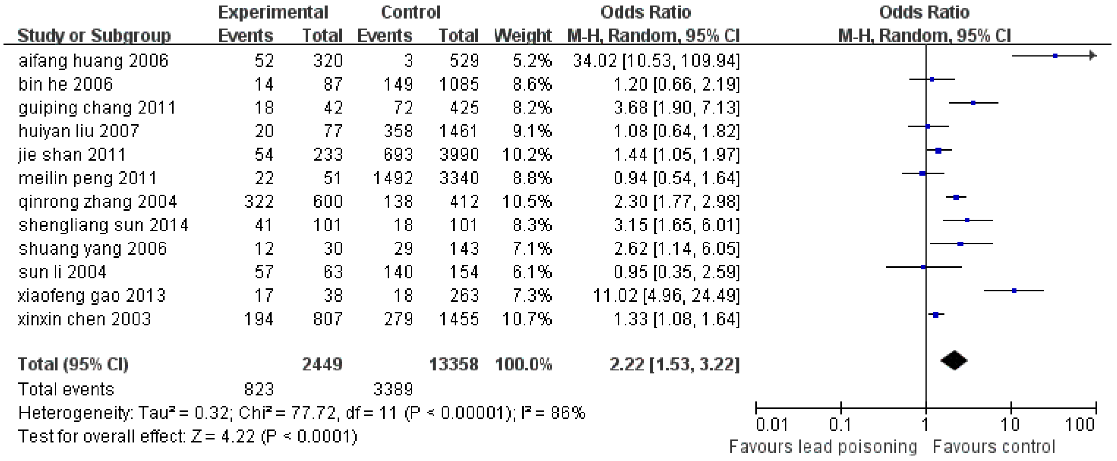

(**b**)

Study or Subgroup	Experimental Events	Experimental Total	Control Events	Control Total	Weight	Odds Ratio M-H, Random, 95% CI
aifang huang 2006	113	320	25	529	8.9%	11.01 [6.93, 17.48]
guiping chang 2011	24	42	158	415	8.1%	2.17 [1.14, 4.12]
hong tian 2006	183	459	161	596	9.6%	1.79 [1.38, 2.32]
hongzhong zhang 2004	233	418	67	120	9.1%	1.00 [0.66, 1.50]
huiyan liu 2007	53	81	918	1492	8.9%	1.18 [0.74, 1.89]
jianghong liu 2012	9	105	86	1239	7.7%	1.26 [0.61, 2.58]
shengliang sun 2014	49	101	22	101	8.2%	3.38 [1.83, 6.24]
shuang yang 2006	16	30	65	143	7.4%	1.37 [0.62, 3.02]
xiaofeng gao 2013	11	38	6	263	6.1%	17.45 [5.98, 50.92]
xiaohua liu 2008	66	120	11	27	7.1%	1.78 [0.76, 4.15]
xiping ma 2005	158	316	287	702	9.6%	1.45 [1.11, 1.89]
zhenyan gao 2014	33	179	329	1849	9.2%	1.04 [0.70, 1.55]
**Total (95% CI)**		**2209**		**7476**	**100.0%**	**2.10 [1.38, 3.20]**
Total events	948		2135			

Heterogeneity: Tau² = 0.46; Chi² = 103.17, df = 11 (P < 0.00001); I² = 89%
Test for overall effect: Z = 3.46 (P = 0.0005)

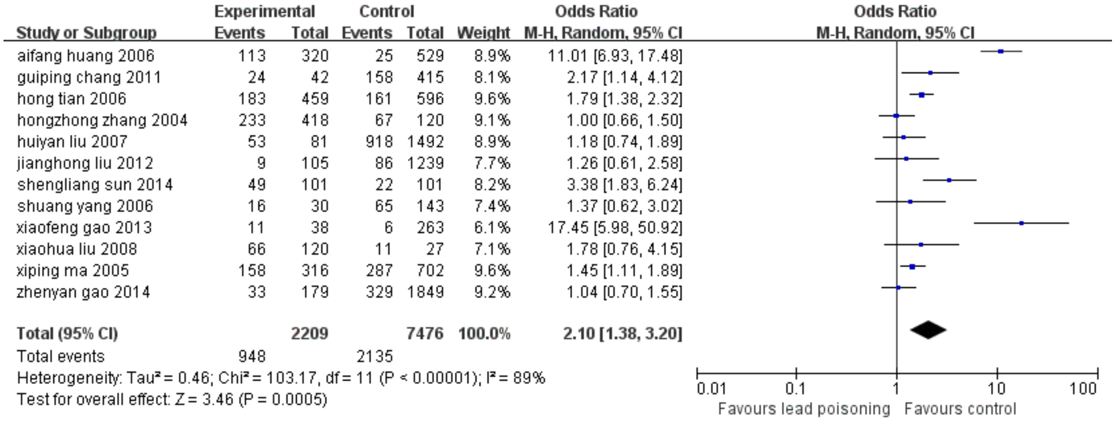

(**c**)

Study or Subgroup	Experimental Events	Experimental Total	Control Events	Control Total	Weight	Odds Ratio M-H, Random, 95% CI
aifang huang 2006	163	320	28	529	4.8%	18.58 [11.97, 28.82]
cangwen tan 2011	270	5295	2512	64673	5.1%	1.33 [1.17, 1.51]
guiping chang 2011	18	42	90	415	4.6%	2.71 [1.41, 5.21]
guo pi 2014	39	165	139	658	4.9%	1.16 [0.77, 1.73]
hong tian 2006	315	459	206	596	5.0%	4.14 [3.19, 5.37]
hongzhong zhang 2004	18	418	7	120	4.2%	0.73 [0.30, 1.78]
huiyan liu 2007	5	82	6	1465	3.6%	15.79 [4.71, 52.89]
jiazheng xu 2007	9	274	27	1997	4.4%	2.48 [1.15, 5.33]
qinrong zhang 2004	476	600	112	412	5.0%	10.28 [7.67, 13.79]
shengliang sun 2014	77	101	50	101	4.6%	3.27 [1.79, 5.97]
shiqiong wang 2010	16	96	59	197	4.6%	0.47 [0.25, 0.87]
shuang yang 2006	1	17	3	101	2.0%	2.04 [0.20, 20.86]
shuwei zhang 2007	313	523	168	1193	5.0%	9.09 [7.16, 11.55]
xiaofeng gao 2013	13	38	9	263	4.1%	14.68 [5.71, 37.72]
xiaozhen xiao 2006	31	41	61	161	4.4%	5.08 [2.33, 11.09]
xiping ma 2005	35	316	32	702	4.8%	2.61 [1.58, 4.30]
xiulan ma 2012	81	153	680	1357	5.0%	1.12 [0.80, 1.57]
xizheng ouyang 2003	194	301	482	823	5.0%	1.28 [0.98, 1.69]
yan yi 2005	26	69	8	61	4.2%	4.01 [1.65, 9.74]
yinyun long 2013	187	239	1860	5612	5.0%	7.25 [5.31, 9.91]
zhenyan gao 2014	100	179	919	1849	5.0%	1.28 [0.94, 1.74]
zhong chen 2015	23	592	216	7590	4.8%	1.38 [0.89, 2.14]
**Total (95% CI)**		**10320**		**90875**	**100.0%**	**2.99 [1.95, 4.59]**
Total events	2410		7674			

Heterogeneity: Tau² = 0.93; Chi² = 581.00, df = 21 (P < 0.00001); I² = 96%
Test for overall effect: Z = 5.03 (P < 0.00001)

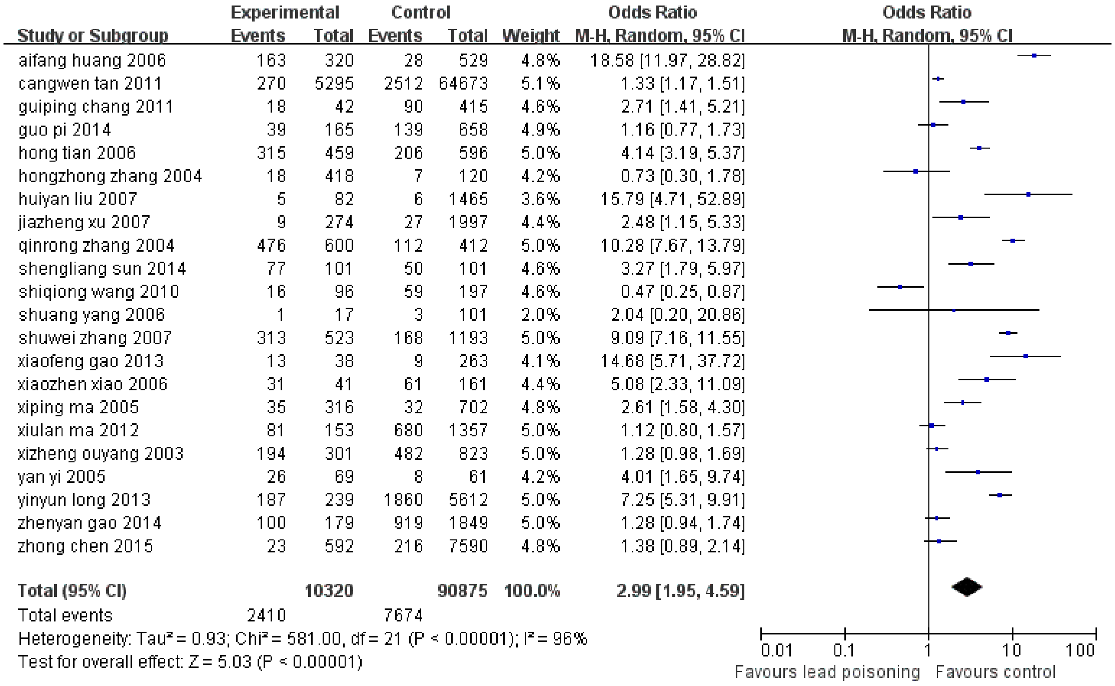

(**d**)

Study or Subgroup	Experimental Events	Experimental Total	Control Events	Control Total	Weight	Odds Ratio M-H, Random, 95% CI
guo pi 2014	96	163	415	627	15.4%	0.73 [0.51, 1.04]
meilin peng 2011	39	51	2232	3343	6.7%	1.62 [0.84, 3.10]
shuang yang 2006	12	17	64	100	2.6%	1.35 [0.44, 4.14]
xinxin chen 2003	509	807	1055	1455	25.4%	0.65 [0.54, 0.78]
zangwen tan 2011	3306	5295	42342	64673	32.5%	0.88 [0.83, 0.93]
zhenyan gao 2014	108	179	1163	1849	17.4%	0.90 [0.66, 1.23]
**Total (95% CI)**		**6512**		**72047**	**100.0%**	**0.84 [0.69, 1.01]**
Total events	4070		47271			

Heterogeneity: Tau² = 0.03; Chi² = 14.67, df = 5 (P = 0.01); I² = 66%
Test for overall effect: Z = 1.88 (P = 0.06)

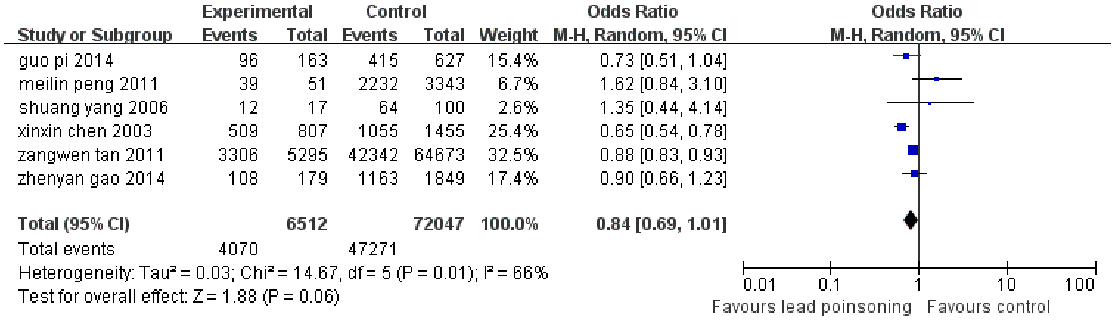

(**e**)

Study or Subgroup	Experimental Events	Experimental Total	Control Events	Control Total	Weight	Odds Ratio M-H, Fixed, 95% CI
shuang yang 2006	2	17	15	100	0.2%	0.76 [0.16, 3.65]
zangwen tan 2011	741	5295	11261	64673	86.3%	0.77 [0.71, 0.84]
zhenyan gao 2014	67	179	656	1849	4.3%	1.09 [0.79, 1.49]
zhong chen 2015	91	592	1284	7590	9.3%	0.89 [0.71, 1.12]
**Total (95% CI)**		**6083**		**74212**	**100.0%**	**0.80 [0.74, 0.86]**
Total events	901		13216			

Heterogeneity: Chi² = 5.23, df = 3 (P = 0.16); I² = 43%
Test for overall effect: Z = 6.07 (P < 0.00001)

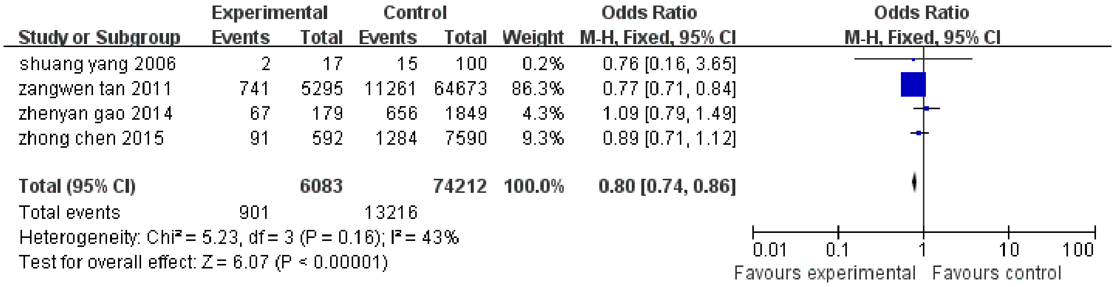

(**f**)

Study or Subgroup	Experimental Events	Experimental Total	Control Events	Control Total	Weight	Odds Ratio M-H, Random, 95% CI
aifang huang 2006	32	320	4	529	4.5%	14.58 [5.11, 41.65]
ailan gou2010	98	158	64	158	7.1%	2.40 [1.53, 3.77]
bin he 2006	10	87	101	1172	6.1%	1.38 [0.69, 2.74]
huiyan liu 2007	21	83	351	1518	6.9%	1.13 [0.68, 1.87]
jianghong liu 2012	42	97	472	1132	7.3%	1.07 [0.70, 1.62]
jie shan 2011	66	233	581	3990	7.7%	2.32 [1.72, 3.12]
meilin peng 2011	14	51	860	3335	6.4%	1.09 [0.59, 2.02]
ning jin 2015	315	661	60	339	7.7%	4.23 [3.08, 5.82]
qi ye 2001	3	9	2	91	2.1%	22.25 [3.10, 159.70]
shengliang sun 2014	49	101	22	101	6.4%	3.38 [1.83, 6.24]
shuang yang 2006	9	30	34	143	5.3%	1.37 [0.58, 3.28]
sun li 2004	40	63	43	154	6.4%	4.49 [2.41, 8.36]
xiaohua liu 2008	59	120	7	27	5.0%	2.76 [1.09, 7.02]
xiaozhen xiao 2006	25	41	41	161	5.9%	4.57 [2.22, 9.40]
xiping ma 2005	53	316	112	702	7.5%	1.06 [0.74, 1.52]
zhenyan gao 2014	65	179	587	1849	7.7%	1.23 [0.89, 1.69]
**Total (95% CI)**		**2549**		**15401**	**100.0%**	**2.26 [1.62, 3.15]**
Total events	901		3341			

Heterogeneity: Tau² = 0.35; Chi² = 97.60, df = 15 (P < 0.00001); I² = 85%
Test for overall effect: Z = 4.81 (P < 0.00001)

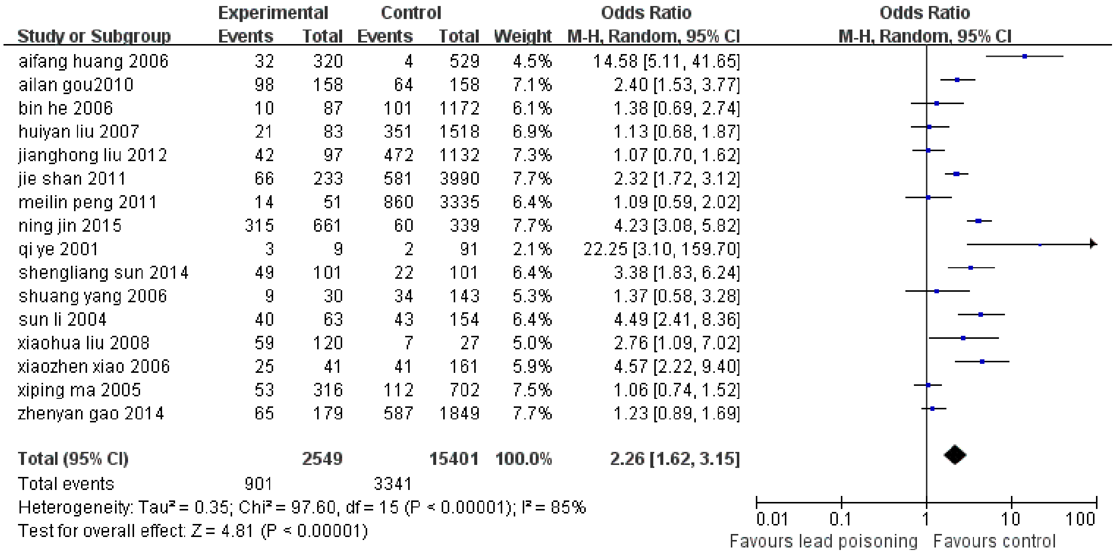

(**g**)

Study or Subgroup	Experimental Events	Experimental Total	Control Events	Control Total	Weight	Odds Ratio M-H, Random, 95% CI
bin he 2006	5	87	38	1172	8.9%	1.82 [0.70, 4.75]
huiyan liu 2007	6	83	59	1518	9.8%	1.93 [0.81, 4.60]
jianghong liu 2012	23	96	347	1142	14.7%	0.72 [0.44, 1.17]
jie shan 2011	72	233	496	3990	17.2%	3.15 [2.35, 4.22]
meilin peng 2011	7	51	354	3340	10.5%	1.34 [0.60, 3.00]
qinrong zhang 2004	349	600	212	412	17.7%	1.31 [1.02, 1.69]
shuang yang 2006	2	30	6	143	4.3%	1.63 [0.31, 8.50]
zhenyan gao 2014	65	179	527	1849	16.9%	1.43 [1.04, 1.97]
**Total (95% CI)**		**1359**		**13566**	**100.0%**	**1.53 [1.04, 2.26]**
Total events	529		2039			

Heterogeneity: Tau² = 0.21; Chi² = 34.52, df = 7 (P < 0.0001); I² = 80%
Test for overall effect: Z = 2.15 (P = 0.03)

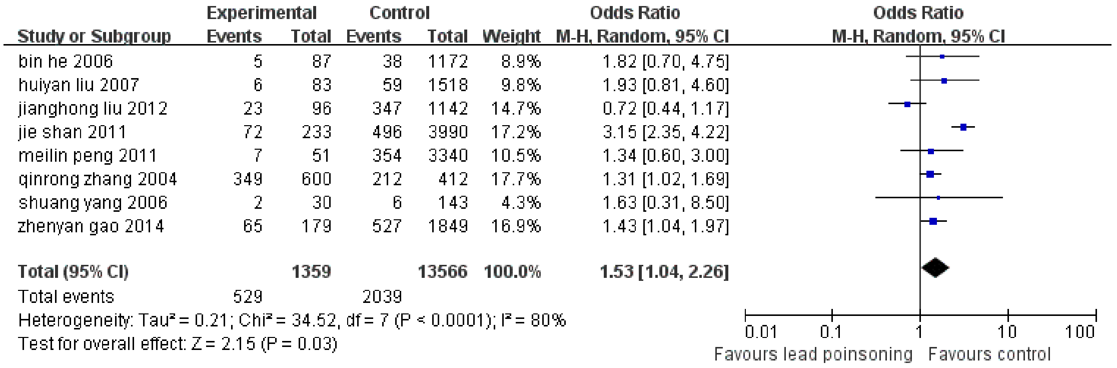

(**h**)

Study or Subgroup	Experimental Events	Experimental Total	Control Events	Control Total	Weight	Odds Ratio M-H, Fixed, 95% CI
ailan gou2010	134	158	146	158	0.6%	0.46 [0.22, 0.95]
bin he 2006	26	87	421	1172	1.2%	0.76 [0.47, 1.22]
guo pi 2014	16	165	113	658	1.2%	0.52 [0.30, 0.90]
jianghong liu 2012	11	105	245	1239	1.0%	0.47 [0.25, 0.90]
jie shan 2011	71	233	1360	3990	3.0%	0.85 [0.64, 1.13]
shuang yang 2006	11	30	67	143	0.4%	0.66 [0.29, 1.48]
xianxiang feng 2006	79	748	225	1163	4.5%	0.49 [0.37, 0.65]
xiaohua liu 2008	68	120	21	27	0.4%	0.37 [0.14, 0.99]
xiulan ma 2012	60	153	718	1357	2.5%	0.57 [0.41, 0.81]
zangwen tan 2011	2234	5295	33631	64673	84.6%	0.67 [0.64, 0.71]
zhenyan gao 2014	5	179	94	1849	0.5%	0.54 [0.22, 1.34]
**Total (95% CI)**		**7273**		**76429**	**100.0%**	**0.66 [0.63, 0.70]**
Total events	2715		37041			

Heterogeneity: Chi² = 12.95, df = 10 (P = 0.23); I² = 23%
Test for overall effect: Z = 15.38 (P < 0.00001)

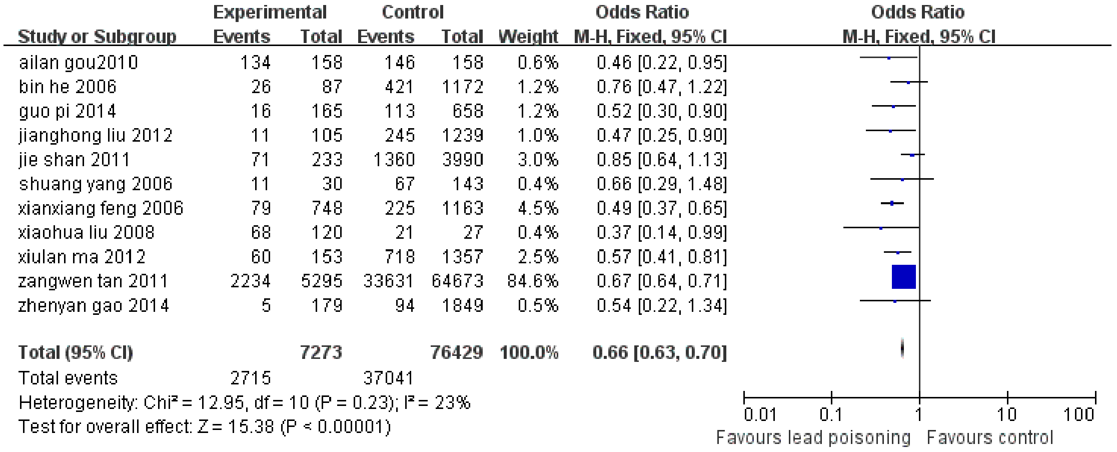

(**i**)

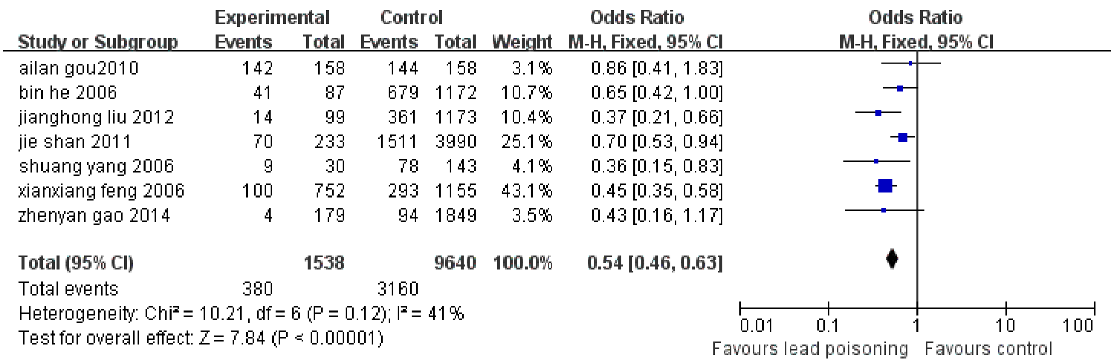
Experimental Control Odds Ratio Odds Ratio
Study or Subgroup Events Total Events Total Weight M-H, Fixed, 95% CI M-H, Fixed, 95% CI
ailan gou2010 142 158 144 158 3.1% 0.86 [0.41, 1.83]
bin he 2006 41 87 679 1172 10.7% 0.65 [0.42, 1.00]
jianghong liu 2012 14 99 361 1173 10.4% 0.37 [0.21, 0.66]
jie shan 2011 70 233 1511 3990 25.1% 0.70 [0.53, 0.94]
shuang yang 2006 9 30 78 143 4.1% 0.36 [0.15, 0.83]
xianxiang feng 2006 100 752 293 1155 43.1% 0.45 [0.35, 0.58]
zhenyan gao 2014 4 179 94 1849 3.5% 0.43 [0.16, 1.17]
Total (95% CI) 1538 9640 100.0% 0.54 [0.46, 0.63]
Total events 380 3160
Heterogeneity: Chi² = 10.21, df = 6 (P = 0.12); I² = 41%
Test for overall effect: Z = 7.84 (P < 0.00001)
0.01 0.1 1 10 100
Favours lead poisoning Favours control

(j)

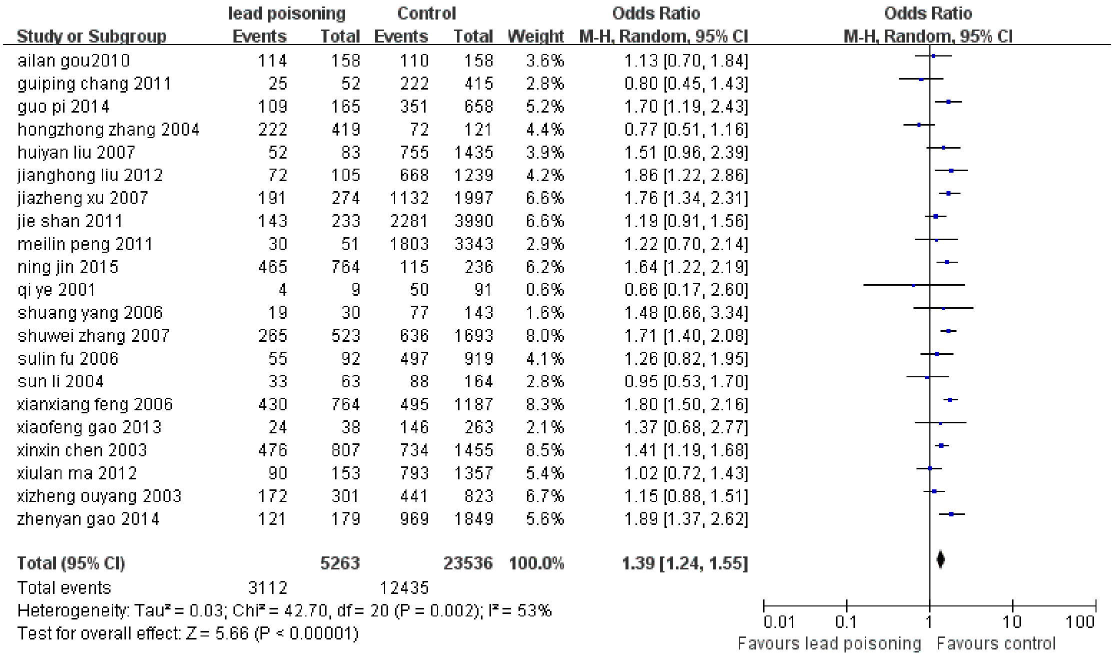
lead poisoning Control Odds Ratio Odds Ratio
Study or Subgroup Events Total Events Total Weight M-H, Random, 95% CI M-H, Random, 95% CI
ailan gou2010 114 158 110 158 3.6% 1.13 [0.70, 1.84]
guiping chang 2011 25 52 222 415 2.8% 0.80 [0.45, 1.43]
guo pi 2014 109 165 351 658 5.2% 1.70 [1.19, 2.43]
hongzhong zhang 2004 222 419 72 121 4.4% 0.77 [0.51, 1.16]
huiyan liu 2007 52 83 755 1435 3.9% 1.51 [0.96, 2.39]
jianghong liu 2012 72 105 668 1239 4.2% 1.86 [1.22, 2.86]
jiazheng xu 2007 191 274 1132 1997 6.6% 1.76 [1.34, 2.31]
jie shan 2011 143 233 2281 3990 6.6% 1.19 [0.91, 1.56]
meilin peng 2011 30 51 1803 3343 2.9% 1.22 [0.70, 2.14]
ning jin 2015 465 764 115 236 6.2% 1.64 [1.22, 2.19]
qi ye 2001 4 9 50 91 0.6% 0.66 [0.17, 2.60]
shuang yang 2006 19 30 77 143 1.6% 1.48 [0.66, 3.34]
shuwei zhang 2007 265 523 636 1693 8.0% 1.71 [1.40, 2.08]
sulin fu 2006 55 92 497 919 4.1% 1.26 [0.82, 1.95]
sun li 2004 33 63 88 164 2.8% 0.95 [0.53, 1.70]
xianxiang feng 2006 430 764 495 1187 8.3% 1.80 [1.50, 2.16]
xiaofeng gao 2013 24 38 146 263 2.1% 1.37 [0.68, 2.77]
xinxin chen 2003 476 807 734 1455 8.5% 1.41 [1.19, 1.68]
xiulan ma 2012 90 153 793 1357 5.4% 1.02 [0.72, 1.43]
xizheng ouyang 2003 172 301 441 823 6.7% 1.15 [0.88, 1.51]
zhenyan gao 2014 121 179 969 1849 5.6% 1.89 [1.37, 2.62]
Total (95% CI) 5263 23536 100.0% 1.39 [1.24, 1.55]
Total events 3112 12435
Heterogeneity: Tau² = 0.03; Chi² = 42.70, df = 20 (P = 0.002); I² = 53%
Test for overall effect: Z = 5.66 (P < 0.00001)
0.01 0.1 1 10 100
Favours lead poisoning Favours control

(k)

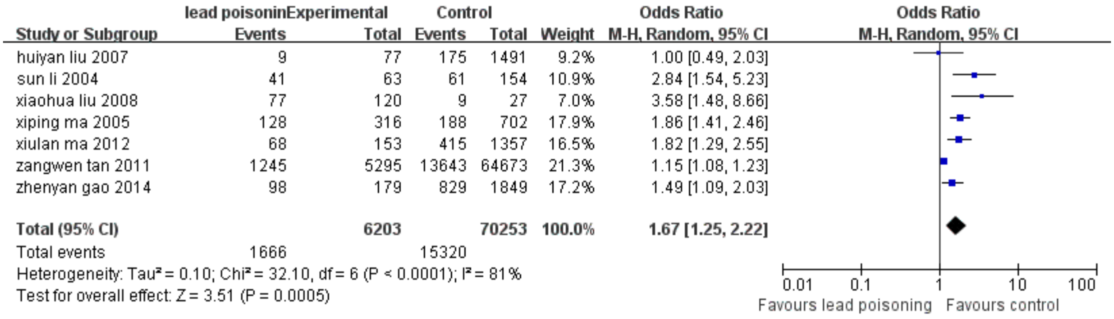
lead poisoninExperimental Control Odds Ratio Odds Ratio
Study or Subgroup Events Total Events Total Weight M-H, Random, 95% CI M-H, Random, 95% CI
huiyan liu 2007 9 77 175 1491 9.2% 1.00 [0.49, 2.03]
sun li 2004 41 63 61 154 10.9% 2.84 [1.54, 5.23]
xiaohua liu 2008 77 120 9 27 7.0% 3.58 [1.48, 8.66]
xiping ma 2005 128 316 188 702 17.9% 1.86 [1.41, 2.46]
xiulan ma 2012 68 153 415 1357 16.5% 1.82 [1.29, 2.55]
zangwen tan 2011 1245 5295 13643 64673 21.3% 1.15 [1.08, 1.23]
zhenyan gao 2014 98 179 829 1849 17.2% 1.49 [1.09, 2.03]
Total (95% CI) 6203 70253 100.0% 1.67 [1.25, 2.22]
Total events 1666 15320
Heterogeneity: Tau² = 0.10; Chi² = 32.10, df = 6 (P < 0.0001); I² = 81%
Test for overall effect: Z = 3.51 (P = 0.0005)
0.01 0.1 1 10 100
Favours lead poisoning Favours control

(l)

Study or Subgroup	Experimental Events	Experimental Total	Control Events	Control Total	Weight	Odds Ratio M-H, Random, 95% CI
guo pi 2014	17	165	65	658	7.1%	1.05 [0.60, 1.84]
hongzhong zhang 2004	111	418	23	120	7.6%	1.52 [0.92, 2.52]
huiyan liu 2007	42	83	699	1518	8.1%	1.20 [0.77, 1.87]
jiazheng xu 2007	48	274	166	1978	8.9%	2.32 [1.63, 3.29]
qi ye 2001	4	9	17	91	2.5%	3.48 [0.84, 14.36]
shengliang sun 2014	73	101	45	101	6.9%	3.24 [1.81, 5.83]
shuang yang 2006	5	17	26	102	3.5%	1.22 [0.39, 3.79]
sulin fu 2006	24	92	246	919	7.7%	0.97 [0.59, 1.57]
xiaofeng gao 2013	14	38	19	263	5.2%	7.49 [3.34, 16.80]
xiaozhen xiao 2006	30	41	73	161	5.6%	3.29 [1.54, 7.01]
xiping ma 2005	123	316	159	702	9.4%	2.18 [1.63, 2.90]
xizheng ouyang 2003	153	301	362	823	9.6%	1.32 [1.01, 1.72]
zangwen tan 2011	1722	5295	19858	64673	10.6%	1.09 [1.02, 1.15]
zhenyan gao 2014	16	179	178	1849	7.3%	0.92 [0.54, 1.58]
**Total (95% CI)**		**7329**		**73958**	**100.0%**	**1.68 [1.29, 2.17]**
Total events	2382		21936			

Heterogeneity: $Tau^2 = 0.16$; $Chi^2 = 83.04$, df = 13 (P < 0.00001); $I^2 = 84\%$
Test for overall effect: Z = 3.91 (P < 0.0001)

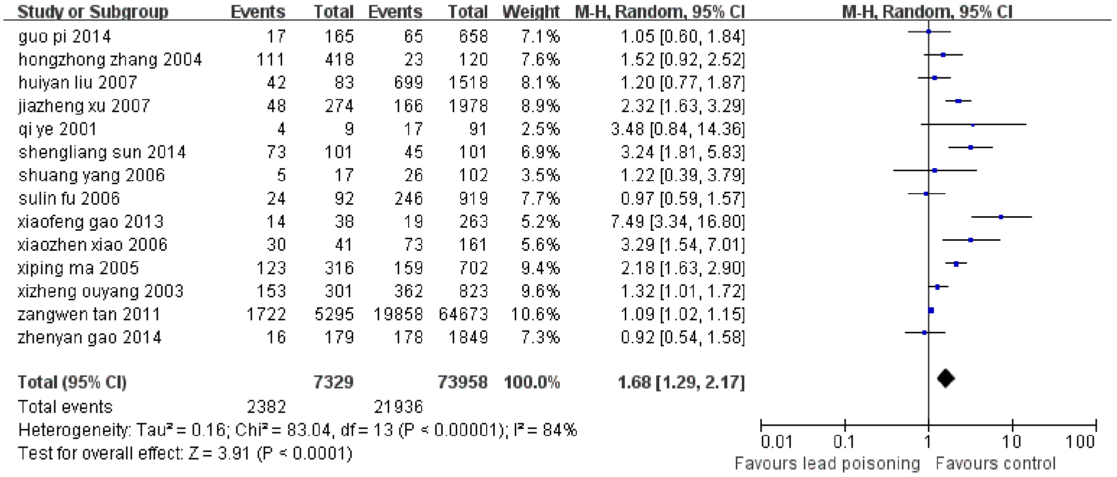

(**m**)

Study or Subgroup	Experimental Events	Experimental Total	Control Events	Control Total	Weight	Odds Ratio M-H, Fixed, 95% CI
shuang yang 2006	0	17	3	102	0.0%	0.81 [0.04, 16.42]
guo pi 2014	85	165	345	658	3.0%	0.96 [0.69, 1.36]
zhenyan gao 2014	12	179	107	1849	0.8%	1.17 [0.63, 2.17]
zangwen tan 2011	1893	5295	20393	64673	89.7%	1.21 [1.14, 1.28]
meilin peng 2011	16	51	917	3343	0.9%	1.21 [0.67, 2.20]
xiping ma 2005	123	316	232	702	4.0%	1.29 [0.98, 1.70]
huiyan liu 2007	1	83	12	1518	0.1%	1.53 [0.20, 11.91]
xiulan ma 2012	15	153	78	1357	0.6%	1.78 [1.00, 3.18]
xiaohua liu 2008	56	120	8	27	0.3%	2.08 [0.84, 5.11]
shengliang sun 2014	73	101	45	101	0.6%	3.24 [1.81, 5.83]
**Total (95% CI)**		**6480**		**74330**	**100.0%**	**1.22 [1.16, 1.29]**
Total events	2274		22140			

Heterogeneity: $Chi^2 = 15.91$, df = 9 (P = 0.07); $I^2 = 43\%$
Test for overall effect: Z = 7.09 (P < 0.00001)

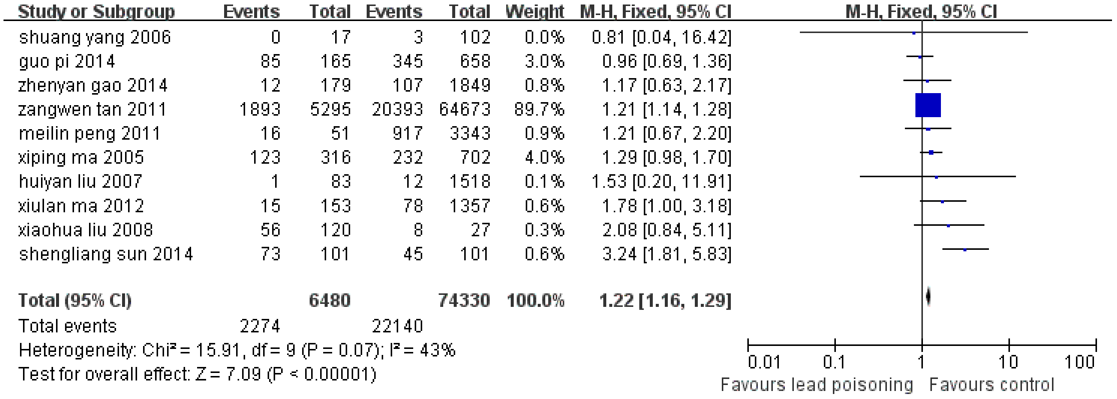

(**n**)

Study or Subgroup	Experimental Events	Experimental Total	Control Events	Control Total	Weight	Odds Ratio M-H, Fixed, 95% CI
hong tian 2006	378	459	356	596	45.9%	3.15 [2.35, 4.21]
hongzhong zhang 2004	39	418	5	120	5.9%	2.37 [0.91, 6.14]
shuang yang 2006	4	17	17	102	3.1%	1.54 [0.45, 5.29]
sulin fu 2006	4	92	40	919	5.8%	1.00 [0.35, 2.86]
xiaohua liu 2008	73	120	8	27	4.3%	3.69 [1.49, 9.11]
xiping ma 2005	70	316	82	702	33.2%	2.15 [1.51, 3.06]
zhenyan gao 2014	4	179	12	1849	1.7%	3.50 [1.12, 10.96]
**Total (95% CI)**		**1601**		**4315**	**100.0%**	**2.62 [2.15, 3.20]**
Total events	572		520			

Heterogeneity: $Chi^2 = 7.52$, df = 6 (P = 0.28); $I^2 = 20\%$
Test for overall effect: Z = 9.49 (P < 0.00001)

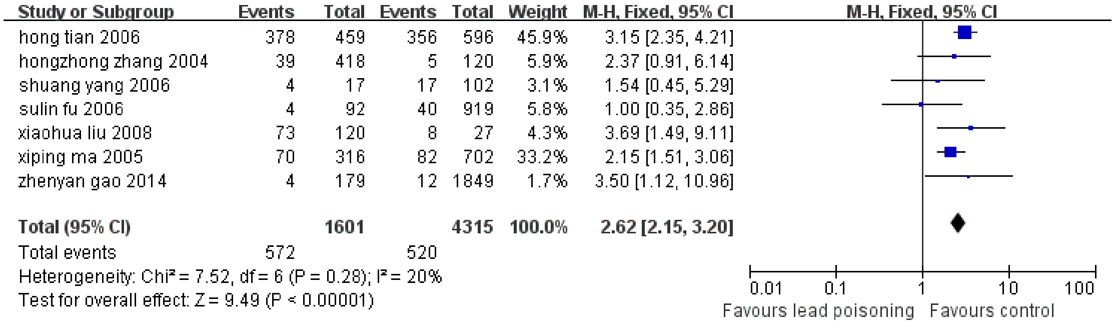

(**o**)

Study or Subgroup	experiment Events	experiment Total	Control Events	Control Total	Weight	Odds Ratio M-H, Random, 95% CI
bin he 2006	8	87	134	1085	7.8%	0.72 [0.34, 1.52]
jiazheng xu 2007	39	274	201	1997	11.5%	1.48 [1.03, 2.14]
jie shan 2011	71	233	1526	3990	12.2%	0.71 [0.53, 0.94]
qinrong zhang 2004	365	600	104	412	12.3%	4.60 [3.49, 6.06]
shuang yang 2006	9	30	20	143	6.5%	2.64 [1.06, 6.57]
xinxin chen 2003	521	807	715	1455	13.0%	1.89 [1.58, 2.25]
xiulan ma 2012	43	153	243	1357	11.4%	1.79 [1.23, 2.62]
zangwen tan 2011	976	5295	10341	69968	13.4%	1.30 [1.21, 1.40]
zhenyan gao 2014	69	179	558	1849	11.9%	1.45 [1.06, 1.99]
**Total (95% CI)**		**7658**		**82256**	**100.0%**	**1.58 [1.14, 2.18]**
Total events	2101		13842			

Heterogeneity: $Tau^2 = 0.20$; $Chi^2 = 114.04$, df = 8 (P < 0.00001); $I^2 = 93\%$
Test for overall effect: Z = 2.77 (P = 0.006)

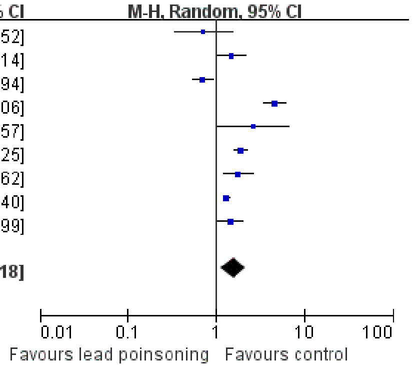

(**p**)

Study or Subgroup	Experimental Events	Experimental Total	Control Events	Control Total	Weight	Odds Ratio M-H, Random, 95% CI
bin he 2006	2	87	46	1085	4.6%	0.53 [0.13, 2.23]
huiyan liu 2007	5	83	44	1516	8.7%	2.14 [0.83, 5.56]
jie shan 2011	57	233	849	3990	24.2%	1.20 [0.88, 1.63]
xinxin chen 2003	137	807	155	1455	26.2%	1.71 [1.34, 2.20]
xiping ma 2005	123	316	159	702	24.9%	2.18 [1.63, 2.90]
zhenyan gao 2014	7	179	107	1849	11.4%	0.66 [0.30, 1.45]
**Total (95% CI)**		**1705**		**10597**	**100.0%**	**1.45 [1.04, 2.02]**
Total events	331		1360			

Heterogeneity: Tau² = 0.09; Chi² = 15.68, df = 5 (P = 0.008); I² = 68%
Test for overall effect: Z = 2.18 (P = 0.03)

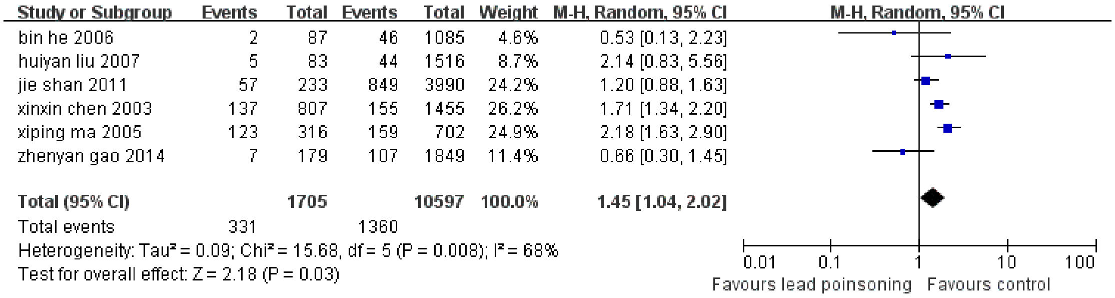

(**q**)

Study or Subgroup	Experimental Events	Experimental Total	Control Events	Control Total	Weight	Odds Ratio M-H, Fixed, 95% CI
bin he 2006	9	87	107	1085	0.9%	1.05 [0.51, 2.16]
jiazheng xu 2007	78	273	408	1971	4.5%	1.53 [1.15, 2.04]
zangwen tan 2011	1142	5295	12484	64673	94.6%	1.15 [1.07, 1.23]
**Total (95% CI)**		**5655**		**67729**	**100.0%**	**1.17 [1.09, 1.25]**
Total events	1229		12999			

Heterogeneity: Chi² = 3.79, df = 2 (P = 0.15); I² = 47%
Test for overall effect: Z = 4.55 (P < 0.00001)

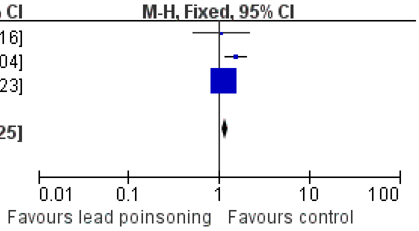

(**r**)

Figure S1: Forest plots for risk factors of child lead poisoning;.(**a**) home painting; (**b**) living near main roads; (**c**) passive smoking; (**d**) often eating foods containing lead; (**e**) frequent consumption of dairy products; (**f**) daily intake of calcium, iron, and/or zinc supplements; (**g**) potential for father's occupational exposure to lead; (**h**) potential for mother's occupational exposure to lead; (**i**) mother's educational level; (**j**) father's educational level;(**k**) sex; (**l**) industry around the home; (**m**) hand-to-mouth activity; (**n**) no often washing hands at keytimes; (**o**) picky eating; (**p**) living on the ground floor; (**q**) coalburning; (**r**) peeling walls.

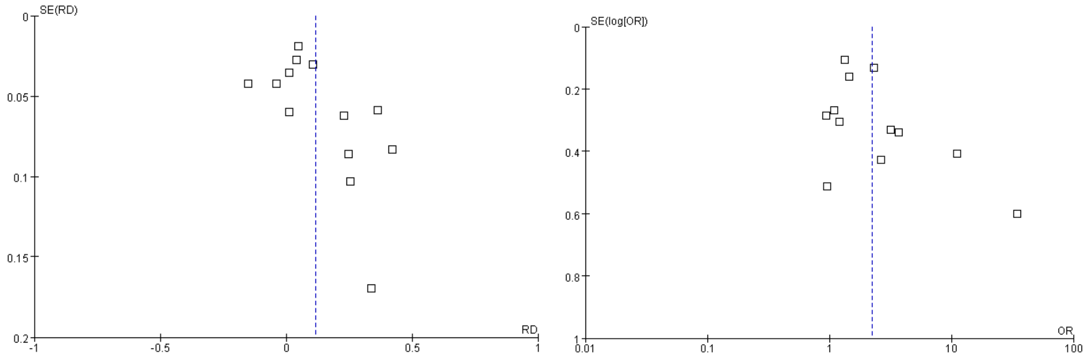

(**a**)

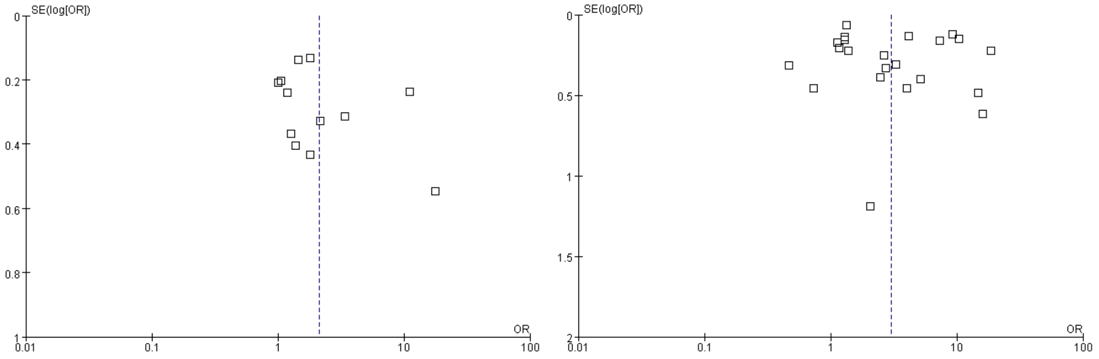

(**b**)

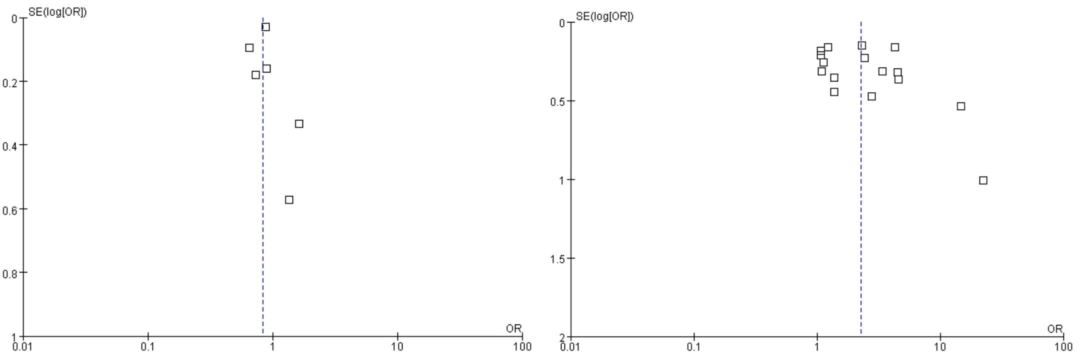
SE(log[OR])
0
0.2
0.4
0.6
0.8
1
OR
0.01
0.1
1
10
100
SE(log[OR])
0
0.5
1
1.5
2
OR
0.01
0.1
1
10
100

(**c**)

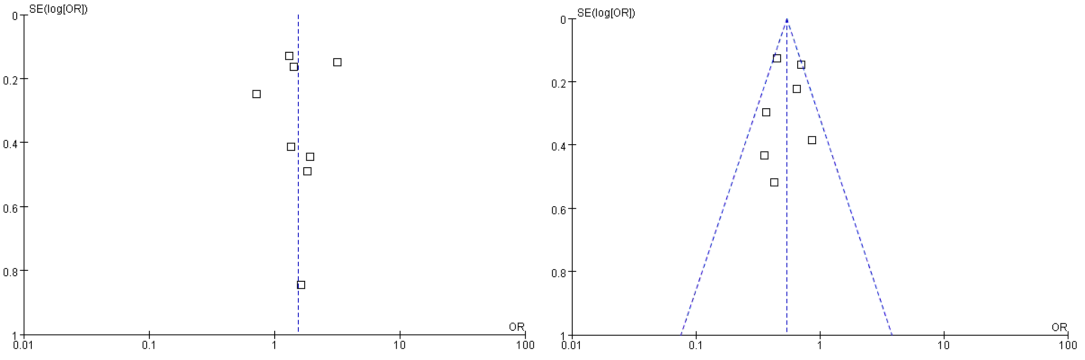
SE(log[OR])
0
0.2
0.4
0.6
0.8
1
OR
0.01
0.1
1
10
100
SE(log[OR])
0
0.2
0.4
0.6
0.8
1
OR
0.01
0.1
1
10
100

(**d**)

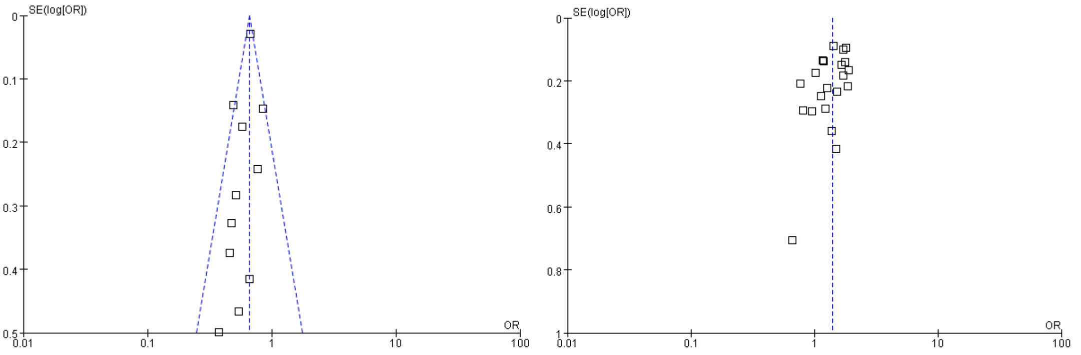
SE(log[OR])
0
0.1
0.2
0.3
0.4
0.5
OR
0.01
0.1
1
10
100
SE(log[OR])
0
0.2
0.4
0.6
0.8
1
OR
0.01
0.1
1
10
100

(**e**)

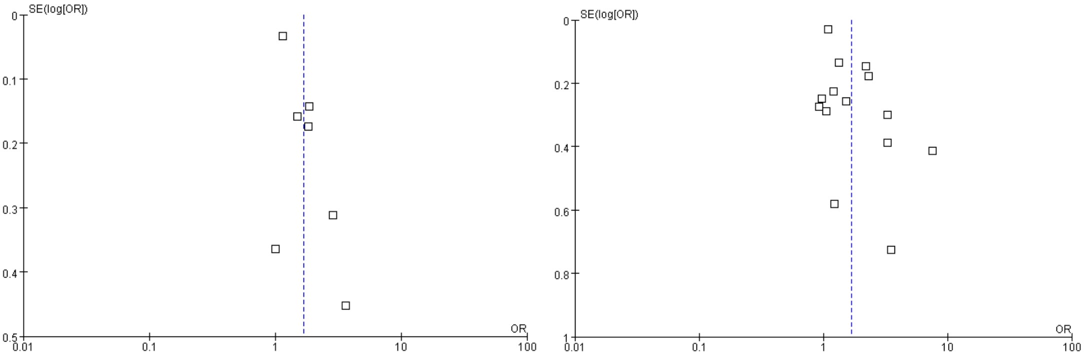
SE(log[OR])
0
0.1
0.2
0.3
0.4
0.5
OR
0.01
0.1
1
10
100
SE(log[OR])
0
0.2
0.4
0.6
0.8
1
OR
0.01
0.1
1
10
100

(**f**)

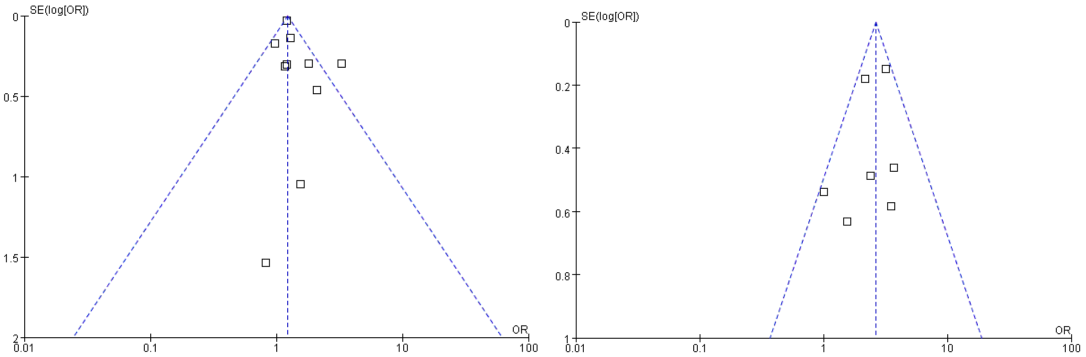

(**g**)

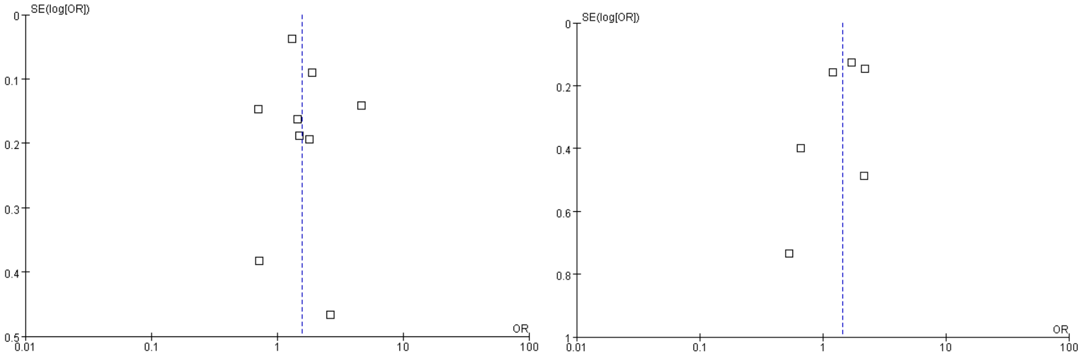

(**h**)

Figure S2. Funnel plots of risk factors for child lead poisoning. (**a**) home painting—living near main roads; (**b**) passive smoking—often eating foods containing lead; (**c**) frequent consumption of dairy products—potential for father's occupational exposure to lead; (**d**) potential for mother's occupational exposure to lead—father's educational level; (**e**) mother's educational level—sex; (**f**) industry around the home—hand-to-mouth activity; (**g**) no often washing hands at keytimes—picky eating; (**h**) living on the ground floor—coalburning.

© 2016 by the authors; licensee MDPI, Basel, Switzerland. This article is an open access article distributed under the terms and conditions of the Creative Commons by Attribution (CC-BY) license (http://creativecommons.org/licenses/by/4.0/).
